# Interproximal wear versus incisors extraction to solve anterior lower crowding: A systematic review

**DOI:** 10.1590/2176-9451.20.1.066-073.oar

**Published:** 2015

**Authors:** Natália Valli de Almeida, Giordani Santos Silveira, Daniele Masterson Tavares Pereira, Claudia Trindade Mattos, José Nelson Mucha

**Affiliations:** 1 Masters student of Orthodontics, Fluminense Federal University (UFF); 2 Specialist in Library science, Integrated Colleges of Jacarepaguá (FIJ); 3 Adjunct professor, Department of Orthodontics, UFF; 4 Full professor, Department of Orthodontics, UFF

**Keywords:** Incisor, Angle Class I malocclusion, Tooth extraction

## Abstract

**OBJECTIVE:**

To determine by means of a systematic review the best treatment, whether interproximal wear or incisor extraction, to correct anterior lower crowding in Class I patients in permanent dentition.

**METHODS:**

A literature review was conducted using MEDLINE, Scopus and Web of Science to retrieve studies published between January 1950 and October 2013. In selecting the sample, the following inclusion criteria were applied: studies involving interproximal wear and/or extraction of mandibular incisors, as well as Class I cases with anterior lower crowding in permanent dentition.

**RESULTS:**

Out of a total of 943 articles found after excluding duplicates, 925 were excluded after abstract analysis. After full articles were read, 13 were excluded by the eligibility criteria and one due to methodological quality; therefore, only fours articles remained: two retrospective and two randomized prospective studies. Data were collected, analyzed and organized in tables.

**CONCLUSION:**

Both interproximal wear and mandibular incisor extraction are effective in treating Class I malocclusion in permanent dentition with moderate anterior lower crowding and pleasant facial profile. There is scant evidence to determine the best treatment option for each case. Clinical decision should be made on an individual basis by taking into account dental characteristics, crowding, dental and oral health, patient's expectations and the use of set-up models.

## Abstract (pt)

**OBJETIVO:**

determinar, por meio de uma revisão sistemática, o melhor tratamento entre desgastes interproximais e extração de incisivos para a correção de apinhamento anteroinferior em pacientes Classe I com dentição permanente.

**MÉTODOS:**

foram feitas buscas nas bases de dados eletrônicas MEDLINE, Scopus e Web of Science por artigos publicados de janeiro de 1950 até outubro de 2013. Os critérios de inclusão foram estudos que abordassem tratamentos com desgastes interproximais e/ou extração de incisivos inferiores, de casos Classe I com apinhamento anteroinferior na dentição permanente.

**RESULTADOS:**

dos 943 artigos encontrados após a remoção dos duplicados, 925 foram excluídos após a leitura dos resumos. Após leitura dos artigos completos, 13 foram excluídos pelos critérios de eligibilidade e um pela qualidade metodológica, restando quatro artigos, sendo dois retrospectivos e dois prospectivos randomizados. Os dados foram coletados, analisados e organizados em tabelas.

**CONCLUSÕES:**

tanto o desgaste interproximal quanto a extração de incisivo inferior são tratamentos eficazes em Classe I na dentição permanente, com apinhamento anteroinferior moderado e perfil facial agradável. Há fracas evidências para determinar a escolha do melhor tratamento para cada caso. A decisão clínica deve ser tomada em bases individuais, considerando as características anatômicas dentárias, da severidade do apinhamento, condições de saúde dentária e bucal, expectativas dos pacientes e ensaio em modelos (*set-up*).

## INTRODUCTION

A pleasant smile and proper alignment of anterior teeth are the main motivation for patients seeking orthodontic treatment.^1^ In permanent dentition, the mandibular anterior region is most susceptible^2^ to patient's dissatisfaction. It is the most common complaint, particularly among older adult patients due to greater exposure of mandibular teeth at smiling.^3^

Orthodontic planning for this type of deficiency may involve permanent teeth extraction^1^^,^ 4 ^-^ 26 or other approaches that do not involve extractions, such as interproximal wear,^6^^-^ 11 ^,^ 14 ^,^ 19 ^,^ 23 ^,^ 24 ^,^ 27 ^-^ 31 dental expansion,^7^^-^ 11 ^,^ 14 distraction osteogenesis of the mandibular symphysis,^32^^,^ 33 as well as a combination of different techniques.^14^

The treatment of choice should be based on a number of features, such as type of malocclusion, negative discrepancy,^17^^,^ 34 facial profile,^8^^,^ 10 ^,^ 11 ^,^ 17 Bolton's ratio,^5^ dental and periodontal conditions,^1^^,^ 5 ^,^ 14 and patient's chief complaint. For a better prognosis, diagnostic,^1^^,^ 5 ^,^ 13 ^,^ 14 ^,^ 19 or virtual set-ups^18^ are indicated.

The aim of this study was to determine - in cases in which there is doubt as to the most appropriate procedure - the best treatment option between interproximal wear and incisor extraction to correct anterior lower crowding in Class I patients in permanent dentition and to achieve good facial esthetics.

## MATERIAL AND METHODS

The guidelines and directives set by the Preferred Reporting Items for Systematic Reviews and Meta-Analysis, the PRISMA Statement, were adopted for this review.^35^

The search, as well as the inclusion/exclusion criteria, were based on PICO format (Table 1).

**Table 1 -:** PICO format.

P = Population	Angle Class I patients in permanent dentition presenting with lower anterior crowding.
I = Intervention	Subjected to orthodontic treatment involving interproximal wear or extraction of a lower incisor.
C = Comparison	Between the two types of treatment and the original characteristics of each malocclusion.
O = Outcome	The best solution for each malocclusion.
Question	What is the best treatment for lower anterior crowding in patients with Class I malocclusion in permanent dentition, interproximal wear or incisor extraction?
Null hypothesis	One treatment is no better than the other.

For sample selection, the following inclusion criteria were applied: studies involving interproximal wear and/or extraction of mandibular incisors in cases of anterior lower crowding and Class I malocclusion in permanent dentition. The exclusion criteria were: case reports; case series; laboratory studies; epidemiological studies; narrative reviews; opinion articles; studies involving orthognathic surgery, distraction osteogenesis, extraction of premolars, syndromic and/or cleft patients, supernumerary teeth and/or abnormal shape of teeth, transverse deficiencies, anterior crossbite, use of auxiliary devices; primary or mixed dentition and/or Class II or III malocclusion.

The literature review was conducted using MEDLINE (via PubMed), Scopus and Web of Science to retrieve studies that met the eligibility criteria and had been published from January 1950 to October 2013, without language restrictions. The combinations of words or terms used are described inTable 2.

**Table 2 -:** List of search parameters used in each database..

Databases	Search parameters
MEDLINE	(wear[tw] OR enamel reduction[tw] OR bolton[tw] OR reproximation[tw] OR reaproximation[tw] OR slenderizing OR tooth wear*[tw] OR tooth wear[MeSH Terms] OR dental wear*[tw] OR dental wear[MeSH Terms] OR tooth attrition[MeSH Terms] OR dental abrasion[MeSH Terms] OR dental abrasion*[tw] OR dental enamel[MeSH Terms] OR dental enamel*[tw] OR non-extraction[tw] OR nonextraction[tw] OR non extraction[tw]) OR (incisor[MeSH Terms] OR incisor*[tw] OR tooth[MeSH Terms] OR tooth[tw] OR teeth[tw] OR tooth extraction*[tw] OR teeth extraction*[tw] OR incisor extraction*[tw] OR extraction*[tw]) AND (tooth crowding[tw] OR tooth crowding[MeSH Terms] OR arch length discrepancy[tw] OR deficiency arch length[tw] OR lower jaw[tw] OR dental irregularity[tw] OR space deficiency[tw] OR lower crowding[tw] OR mandibular crowding[tw] OR incisor crowding[tw] OR crowded[tw]) AND (malocclusion, angle class I[MeSH Terms] OR angle class I[tw]) Filters: ppublication date from 1950/01/01
Scopus	(((ALL(wear) OR ALL(“enamel reduction”) OR ALL(bolton) OR ALL(reproximation) OR ALL(reaproximation) OR ALL(slenderizing) OR ALL(“tooth wear”) OR ALL(“tooth wears”) OR ALL(“dental wear”) OR ALL(“dental wears”) OR ALL(“tooth attrition”) OR ALL(“dental abrasion”) OR ALL(“dental abrasions”) OR ALL(“dental enamel”) OR ALL(“dental enamels”) OR ALL(“non-extraction”) OR ALL(nonextraction) OR ALL(“non extraction”))) OR ((ALL(incisor) OR ALL(incisors) OR ALL(tooth) OR ALL(teeth) OR ALL(“tooth extraction”) OR ALL(“tooth extractions”) OR ALL(“teeth extractions”) OR ALL(“teeth extraction”) OR ALL(“incisor extraction”) OR ALL(“incisor extractions”) OR ALL(extraction) OR ALL(extractions)))) AND ((ALL(“tooth crowding”) OR ALL(“arch length discrepancy”) ORA LL(“deficiency arch length”) OR ALL(“lower jaw”) OR ALL(“dental irregularity”) OR ALL(“space deficiency”) OR ALL(“lower crowding”) OR ALL(“mandibular crowding”) OR ALL(“incisor crowding”) OR ALL(“crowded”))) AND((ALL(“malocclusion angle class I”) OR ALL(“angle class I”) OR ALL(“class I”)))
Web of Science	#1 = TS=(wear) OR TS=(enamel reduction) OR TS=(bolton) OR TS=(reproximation) OR TS=(reaproximation) OR TS=(slenderizing) OR TS=(tooth wear*) OR TS=(dental wear*) OR TS=(tooth attrition) OR TS=(dental abrasion) OR TS=(dental enamel*) OR TS=(non-extraction) OR TS=(non extraction) OR TS=(nonextraction) #2 = TI=(wear) OR TI=(enamel reduction) OR TI=(bolton) OR TI=(reproximation) OR TI=(reaproximation) OR TI=(slenderizing) OR TI=(tooth wear*) OR TI=(dental wear*) OR TI=(tooth attrition) OR TI=(dental abrasion) OR TI=(dental enamel*) OR TI=(non-extraction) OR TI=(non extraction) OR TI=(nonextraction) #3 = TS=(incisor) OR TS=(tooth) OR TS=(teeth) OR TS=(tooth extraction*) OR TS=(teeth extraction*) #4 = TI=(incisor) OR TI=(tooth) OR TI=(teeth) OR TI=(tooth extraction*) OR TI=(teeth extraction*) #5 = TS=(tooth crowding) OR TS=(tooth crowding) OR TS=(arch length discrepancy) OR TS=(deficiency arch length) OR TS=(lower jaw) OR TS=(dental irregularity) OR TS=(space deficiency) OR TS=(lower crowding) OR TS=(mandibular crowding) OR TS=(incisor crowding) OR TS=(crowded) #6 = TI=(tooth crowding) OR TI=(tooth crowding) OR TI=(arch length discrepancy) OR TI=(deficiency arch length) OR TI=(lower jaw) OR TI=(dental irregularity) OR TI=(space deficiency) OR TI=(lower crowding) OR TI=(mandibular crowding) OR TI=(incisor crowding) OR TI=(crowded) #7 TS=(malocclusion angle class I) OR TS=(angle class I) OR TS=(class I) #8 TI=(malocclusion angle class I) OR TI=(angle class I) OR TI=(class I) #1 OR #2 = #9 / #3 OR #4 = #10 / #5 OR #6 = # 11 / #7 OR #8 = #12 / #9 OR #10 = #13 / #13 AND #11 AND #12 Time period covered by searches = 1950-2013

Duplicate articles were eliminated from the final search results. Titles and abstracts were read independently by two reviewers who analyzed the articles in light of the inclusion and exclusion criteria. All articles found to be compatible and somehow related to the question (Table 1) were reviewed. Disagreements between reviewers were settled in a consensus meeting held with a third investigator. The articles selected were fully read. The references of the articles included in the research were also analyzed in search of potential relevant articles that might not have been found in the selected databases.

The articles selected were assessed for methodological quality according to a list based on CONSORT,^36^ whenever applicable, and modified by the reviewers (Table 3). Disagreements were solved in consensus meetings, and articles were classified into high (≥13), moderate (<13 and ≥9) and low (<9) methodological quality.

**Table 3 -:** Methodological quality assessment - based on CONSORT.35

	Methodological quality features assessed in the included studies	Score
A	Description of study objectives	1
B	Study design (retrospective = 0 point; prospective = 1 point; randomized prospective = 2 points)	2
C	Description of sample inclusion/exclusion criteria	1
D	Intervention clearly described (reason for choosing the extracted tooth/performing the wear)	1
E	Measures for evaluating the results described	1
F	Determining the sample size (sample size calculation)	1
G	Description of statistical analysis methods	1
H	Sample description (demographic - age, sex and ethnicity)	1
I	Sample description (overjet, overbite, perimeter discrepancy, Bolton, tooth form, oral health, profile) (0.5 point/item. More than 6 items = 3 points)	3
J	Description of treatment duration and follow-up (1 point each)	2
K	Description of limitations, biases and inaccuracies of the study	1
L	Operator calibration	1

Data were extracted from the articles by two reviewers.

## RESULTS

**Figure 1 -:**
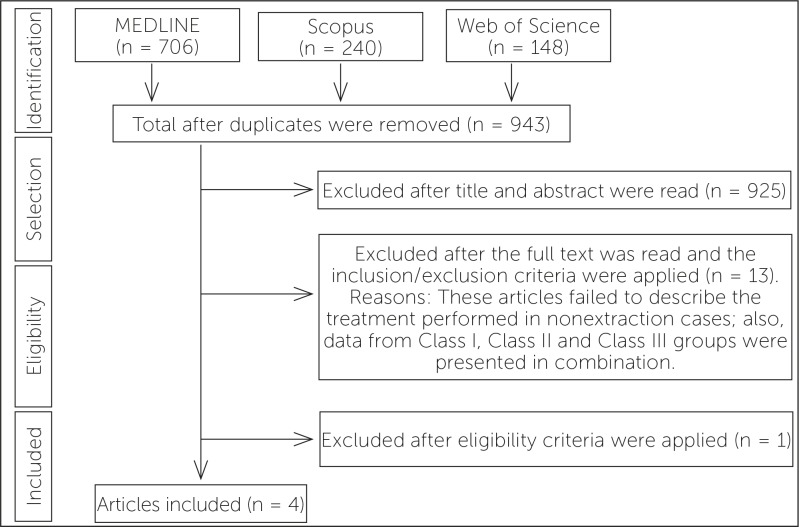
PRISMA flow diagram of database research results.

 The search in the literature identified 1,094 studies, 706 from MEDLINE, 240 from Scopus and 148 from Web of Science, which are all presented in a "Prism Flow Diagram"^35^ (Fig 1). After excluding 151 repeated articles, all titles and abstracts were read and those found to be unrelated to the review were eliminated. Eighteen preselected articles were read in full and the inclusion and exclusion criteria were applied. Five articles remained and were classified according to the methodological quality assessment.

One article was assigned as presenting low methodological quality^22^ and was, therefore, not included in this study. Four articles showed moderate quality,^23^^-^ 26 and none presented high quality (Table 4). Most articles offered insufficient sample description, both demographically and in terms of sample size calculation.

**Table 4 -:** Methodological quality scores for the selected articles. Items A to L are described in Table 3.

Studies	A	B	C	D	E	F	G	H	I	J	K	L	Points	Quality
Dacre^26^	1	0	0.5	0	1	0	1	0.5	2	2	0	1	9	Moderate
Biondi^22^	0	0	0	1	1	0	0	0.5	1	0	0	0	3.5	Low
Germeç et al^23^	1	2	1	1	1	0	1	0.5	2	1	0	1	11.5	Moderate
Germec-Cakan et al^24^	1	2	1	1	1	0	1	0.5	1	1	1	1	11.5	Moderate
Ileri et al^25^	1	0	1	0	1	1	1	0.5	2	1	1	1	10.5	Moderate

Of the four studies included, two were randomized prospective^23^^,^ 24 and two were retrospective studies.^25^^,^ 26 Only one article presented sample size calculation.^25^ In the study by Ileri et al,^25^ only the sample data for incisor extraction (IE) were considered, given that no wear was mentioned in the non extraction (NE) group, and although the authors were contacted by e-mail, no response was given. Only the data from groups of interest were extracted from the articles.^23^^-^ 26

All information regarding the author, year, study type, sample, type of treatment, statistical analysis, data evaluated and total treatment time, was gleaned from the included articles and described inTable 5.

**Table 5 -:** Data obtained from articles included.

	Dacre,^26^ 1985	Germeç et al,^23^ 2008	Germec-Cakan et al,^24^ 2010	Ileri et al,^25^ 2012
Study type	Retrospective	Randomized prospective	Randomized prospective	Retrospective
n / sex	8F/8M	11F/2M	11F/2M	13F/7M
Mean age (years)	15.0 ± 2.7	17.8 ±2.4	17.8 ± 2.4	14.3 ± 2.9
Treatment type	IE	NE = Air rotor wear (AIR) from mesial of 1st molar to mesial of 1st molar	NE = Air rotor wear (AIR) from mesial of 1st molar to mesial of 1st molar	IE
Statistical analysis	Dahlberg’s formula Snedecor’s F ratio T-test	Wilcoxon test Mann-Whitney U test Dahlberg’s formula T-test	Wilcoxon test Mann-Whitney U test Dahlberg’s formula	ANOVA Tukey HSD Mann-Whitney U test
Treatment duration (years)	1.8 ± 1.4	ND	17.0 ± 4.6	1.6 ± 0.9
Author’s conclusion	Overjet and overbite increased mildly after incisor extraction with clinical significance varying from patient to patient. Posterior occlusion was not affected.	In determining treatment for borderline Class I patients the following should be considered: Treatment duration with premolar extraction, AIR limitations (enamel thickness, tooth morphology, convexity of the proximal surface), and in facial changes resulting from growth.	In Class I borderline patients with moderate crowding the extraction of premolars with minimum anchorage does not result in a narrower arch. Furthermore, in treatments without extraction both the intercanine width and the arch perimeter are preserved.	Treatments without extraction yield better results than those involving extraction of 4 first premolars, or extraction of incisors in Class I patients with moderate to severe crowding. Tooth size discrepancy should be considered to ensure satisfactory interdigitation of upper and lower teeth.

F = females; M = males; IE = incisor extraction; NE = nonextraction (interproximal wear); ND = not declared.

**Table 6 -:** Data obtained from articles included.

Author / year	Data assessed			
Dacre,^26^ 1985		T_1_	T_2_	
	SNA	81.7±4.27	82.5±4.41	
	SNB	78.2±3.72	79.1±3.78	
	SNI	82.4±4.36	82.5±4.60	
	Overjet	3.30±.1.27	4.40±1.69	
	Overbite	3.10±1.59	3.90±1.85	
	CD	24.7±1.42	22.5±1.42	
	Crowding Severe Moderate Mild Aligned Space			
	Initial 9 6 1 - -			
	Final - 1 7 5 3			
Germeç et al,^23^ 2008	Crowding (mm)			
	NE = -5.9 ± 1.3			
ARS performed			
Upper: 5.4±1.7 (2.6±0.9 mm ant / 2.8±1.0 mm post)			
Lower: 5.1±0.9 (2.0±0.5 mm ant / 3.1±0.9 mm post)			
	T_1_	T_2_	P
Overjet	3.1±0.8	2.9±0.8	0.578
Overbite	2.4±1.6	3.0±0.9	0.280
Cephalometric measurements			
FMA (^o^)	24.5±3.9	24.3±4.1	0.186
AFI (^o^)	46.4±2.3	46.3±2.4	0.765
SNA (^o^)	79.5±3.6	79.5±2.9	0.821
SNB (^o^)	77.2±2.2	76.9±2.5	0.490
Pog-NB (mm)	2.0±1.6	2.5±2.0	0.027*
IMPA (^o^)	94.9±6.9	88.7±6.3	0.002**
Nasolabial ang (^o^)	108.5±8.9	109.9±10.4	0.366
UL-E-plane (mm)	-5.4±1.7	-6.4±1.8	0.046*
LL-E-plane (mm)	-2.4±1.6	-3.6±2.1	0.013*
L1-NB (^o^)	26.8±4.2	20.9±4.7	0.002**
UL-PTV (mm)	71.1±3.3	71.0±3.5	0.721
LL-PTV (mm)	69.0±4.0	68.9±4.0	0.479
*P < 0.05 **P < 0.01			
Germec-Cakan et al,^24^ 2010	Crowding			
	NE = -5.9 ± 1.3			
		T_1_	T_2_	P
	CD upper	34.02±2.98	33.78±2.04	0.78
	MD upper	50.49±2.79	49.42±2.13	0.011*
	P upper	75.46±4.91	75.15±3.36	0.469
	CD lower	24.60±2.25	25.52±1.45	0.173
	MD lower	43.07±3.29	41.81±2.34	0.046*
	P lower	63.46±3.91	64.15±3.05	0.214
	*P < 0.05			
Ileri et al,^25^ 2012		Mean ± SD	ANOVA	
	PAR %	80.3±18	*(P < 0.05)	
	Anterior ratio	81.7±4.5	***(P < 0.01)	
	Overall ratio	94.2±2.9	**(P < 0.001)	
	PAR score	T_1_	T_2_	
		21.5±11.5	3.8±3.52	

T1 = pretreatment; T2 = post-treatment; PAR% = PAR index = T2-T1 x 100/PAR T1; MD = intermolar distance; CD = intercanine distance; P = arch perimeter.

Data analyzed in each study varied widely. Ileri et al^25^ assessed changes in the PAR index and Bolton ratio, and treatment included mandibular incisor extraction. Dacre^26^ correlated cephalometric measurements, overjet, overbite and initial intercanine width also involving mandibular incisor extraction. Germeç et al^23^ analyzed the effect of interproximal wear on cephalometric measurements, overbite and overjet. Germec-Cakan et al^24^ compared intercanine and intermolar widths, as well as pre and post-treatment arch perimeter after interproximal wear. Only one study^26^ described sample follow-up. Three studies^24^^,^ 25 ^,^ 26 mentioned treatment time.

Given that studies included different data, it was impossible to compare them directly and/or perform meta-analysis.

## DISCUSSION

By the end of this research, only one systematic review^37^ with indications, contraindications and effects of extracting a mandibular incisor in patients with different malocclusions, was found. Our review, however, had a different goal: to determine the advantages and disadvantages as well as the indications and contraindications of interproximal wear *versus* incisor extraction for correction of anterior lower crowding in patients in permanent dentition and Class I malocclusion.

Several clinical cases^1^^,^ 2 ^,^ 5 ^,^ 9 ^,^ 12 ^-^ 15 ^,^ 17 ^-^ 21 ^,^ 30 ^,^ 31 ^,^ 38 reported interproximal wear or mandibular incisor extraction as potential therapies for mild or moderate anterior lower crowding in patients in permanent dentition, with Class I malocclusion and a pleasant facial profile. Nevertheless, there are yet few clinical trials or randomized controlled trials addressing this issue.

Of the 943 articles found after duplicates removal, only eighteen were selected for full reading. The articles excluded after title and abstract reading included case reports or epidemiological research. Either that or the sample had undergone treatment for crossbite, distal movement of molars, surgical treatment and extraction of other permanent teeth. Some articles addressed mixed and primary dentition, or only Class II or Class III malocclusion.

Of the eighteen^16^^,^ 22 ^-^ 26 ^,^ 37 ^,^ 39 ^-^ 49 articles included for full reading, only five^22^^-^ 26 were selected for methodological quality assessment. The reasons for exclusion were: no description of treatment used when referring to nonextraction; lack of clear information on whether or not interproximal wear had been performed; treatment including dental arch expansion or incisor protrusion;^39^^,^ 40 ^,^ 42 ^-^ 49 use of auxiliary appliances;^40^ systematic review performed using some other approach;^37^ description of clinical cases;^16^ and whenever data from Class I, II and III groups were presented together, which precluded the use of data from Class I patients, only.^41^

Only one^22^ out of the five articles selected for methodological assessment was excluded due to low methodological quality and also because it failed to report the final results. Two out of the four articles included after qualifying addressed treatment with incisor extraction^25^^,^ 26 while two reported using interproximal wear.^23^^,^ 24

Mandibular wear performed in the study by Germeç et al^23^ measured 5.1 ± 0.9 mm, with 2.0 ± 0.5 mm in anterior lower teeth, only. To solve crowding of 4 mm to 8 mm, Sheridan^50^ advocates interproximal reduction carried out mostly, but not exclusively, in the anterior segment. Wear should be limited to about 0.5 mm on each side of anterior teeth, and 0.8 mm on posterior teeth.^9^^,^ 28 It should not exceed 50% of total enamel thickness.^7^ The areas of mandibular teeth where enamel thickness is greater are the distal surfaces of lateral incisors^2^^,^ 7 and the mesial and distal surfaces of canines.^2^

Germec-Cakan et al^24^ observed that cases in which interproximal wear was carried out had a decrease in intermolar width whereas intercanine width and arch perimeter remained unchanged. This treatment allows the creation of a contact area between teeth, which favors stability.^6^ When performed carefully, interproximal wear yields a healthy dentition, which is not susceptible to periodontal disease and tooth decay.^29^^,^ 51 There is a certain degree of concern, however, that a thin interdental alveolar septum might accelerate gingival attachment loss and the spread of periodontal disease.^52^

According to Ileri et al,^25^ a PAR index comparison showed that malocclusions were corrected by extracting mandibular incisors, which was indicated in cases with mandibular anterior Bolton^53^ discrepancy whereby the anterior ratio equals to 81.7 ± 4.5,25, thereby corroborating other articles.^5^^,^ 13 ^,^ 16 ^,^ 17 ^,^ 18 ^,^ 25 ^,^ 37 ^,^ 38 ^,^ 54 This seems to suggest that in cases in which mandibular dental volume excess is smaller, the best alternative may be interproximal wear.^15^^,^ 16 The other groups compared by Ileri et al^25^ (premolar extraction and treatment without extraction) were assigned better scores after treatment, perhaps due to difficult intercuspation and/or overjet remaining in cases involving mandibular incisor extraction.^25^ Thus, in these cases, interproximal wear is indicated on maxillary anterior teeth to correct remaining overjet.^1^^,^ 5 Priority should be given to extracting incisors in patients with decreased overjet and overbite.^13^^,^ 16 ^,^ 18 ^,^ 20 ^,^ 37 ^,^ 38

Dacre^26^ showed in a follow-up of 16 patients, after mandibular incisor extraction and retainer removal, that only five cases preserved good alignment, while seven had mild crowding relapse, one had moderate relapse, and three showed space opening. Intercanine width was slightly reduced, since extraction caused canines to move closer to the region where the dental arch is narrower.^26^

Selection of the incisor to be extracted is usually based on malposition, periodontal involvement, color change, decay and/or fracture,^1^^,^ 18 factors which are less likely to induce changes in profile,^5^^,^ 12 and arch length.^13^ Loss of interdental papilla or formation of triangular space are examples of common undesirable effects.^13^^,^ 16 ^,^ 37 From an esthetic point of view, teeth with a triangular shape^2^^,^ 31 may benefit from interproximal wear while those with a rectangular shape respond better to extraction.

Total treatment time was similar between the studies by Ileri et al^25^ and Germec-Cakan et al;^24^ and both were shorter when compared to the group in which premolars were extracted. Other authors also reported decreased treatment time due to incisor extraction.^5^^,^ 14 ^,^ 17 ^,^ 54

Patients with the following characteristics may benefit from mandibular incisor extraction: Bolton's tooth-size discrepancies ≥ 4 mm,^5^^,^ 12 ^,^ 13 ^,^ 16 ^,^ 17 ^,^ 18 ^,^ 25 ^,^ 37 ^,^ 38 ^,^ 54 mild to moderate mandibular crowding,^5^^,^ 13 ^,^ 14 ^,^ 17 ^-^ 21 ^,^ 23 ^,^ 28 ^,^ 29 ^,^ 4 a tendency towards or moderate Class III,^1^^,^ 16 ^,^ 37 Class I,^1^^,^ 12 ^,^ 13 ^,^ 16 ^,^ 17 ^,^ 18 ^,^ 20 ^,^ 25 ^,^ 26 or Class II malocclusion,^55^ a pleasant facial profile,^5^^,^ 12 ^,^ 18 ^,^ 20 decreased overjet and overbite,^13^^,^ 16 ^,^ 18 ^,^ 20 ^,^ 37 ^,^ 38 structurally and periodontally compromised teeth, teeth with a rectangular shape,^1^^,^ 18 ^,^ 19 ^,^ 37 supernumerary incisors,^37^ ectopic eruption,^37^ TMD involving a retropositioned mandible,^37^ mild or nonexistent maxillary crowding,^1^^,^ 16 ^,^ 17 ^,^ 18 ^,^ 20 absence of or abnormality in the shape of maxillary central or lateral incisors,^17^^-^ 20 patients with complete growth,^18^^,^ 20 and treatment confirmed by set-up model tests.^1^^,^ 5 ^,^ 13 ^,^ 14 ^,^ 18 ^,^ 19

Interproximal wear should be given priority when aiming at conservative treatment^2^^,^ 30 with minor changes in a pleasant profile,^2^^,^ 23 ^,^ 30 in Class I cases,^2^^,^ 9 ^,^ 23 ^,^ 24 ^,^ 30 cases without mandibular dental excess (Bolton ≤ 3 mm),^15^^,^ 16 mild to moderate mandibular crowding,^2^^,^ 16 ^,^ 23 ^,^ 24 ^,^ 30 ^,^ 31 normal overjet and overbite, low incidence of caries,^2^ proper oral hygiene,^31^ teeth with a triangular shape,^2^^,^ 31 potential for maxillary wear, and treatment confirmed by set-up model tests.^1^^,^ 5 ^,^ 13 ^,^ 14 ^,^ 18 ^,^ 19

Several case reports^1^^,^ 2 ^,^ 5 ^,^ 9 ^,^ 12 ^-^ 15 ^,^ 17 ^-^ 21 ^,^ 30 ^,^ 31 ^,^ 38 addressing the issue were not included, given their low evidence and inference that these cases were successful. Lack of high-methodological-quality articles is a limitation of the present study. Nevertheless, no studies have been found with good methodological quality comparing the two treatments in patients with Class I malocclusion, moderate crowding and pleasant facial profile. However, there is credible evidence^23^^,^ 24 ^,^ 25 showing that treatment involving interproximal wear and incisor extraction do help to improve malocclusion.

## CONCLUSIONS

» Both mandibular incisor extraction and interproximal wear are effective to treat patients with Class I malocclusion with moderate anterior lower crowding, in permanent dentition and with a pleasant facial profile. There is, however, scant evidence to determine the best treatment approach.

» Decreased overjet, overbite and Bolton's tooth-size discrepancy were the most decisive parameters used to indicate mandibular incisor extraction.

» Clinical decision should be made on an individual basis by taking into account patient's dental anatomical characteristics, crowding, dental and oral health conditions, expectations and the use of set-up models.
